# Recurrent reactive hypoglycemia due to clozapine‐induced glucose intolerance: A case report

**DOI:** 10.1002/pcn5.162

**Published:** 2023-12-21

**Authors:** Fumihiko Kawai, Satsuki Watanabe, Hirotaka Ebihara, Naoki Shimizu, Shiori Oshima, Hiroshi Okai, Yoshiko Murata, Hisatoshi Arai, Koji Matsuo

**Affiliations:** ^1^ Department of Psychiatry, Faculty of Medicine Saitama Medical University Saitama Japan

**Keywords:** diabetes, impaired glucose tolerance, insulin, oral glucose tolerance test, voglibose

## Abstract

**Background:**

Although diabetes is one of the most common side effects of clozapine, a medication for the treatment of schizophrenia, to the best of our knowledge no study exists on clozapine‐induced glucose intolerance or hypoglycemia in patients with schizophrenia.

**Case Presentation:**

We report a case of schizophrenia with repeated reactive hypoglycemia due to abnormal glucose tolerance during clozapine treatment. During clozapine administration in patients with schizophrenia, it is necessary to monitor physical and psychiatric symptoms due to reactive hypoglycemia and hyperglycemia. If abnormal glucose tolerance is a concern, it should be promptly detected using blood or oral glucose tolerance tests.

**Conclusion:**

Early intervention for impaired glucose tolerance may prevent clozapine discontinuation due to diabetes or hyperglycemia.

## Graphical abstract

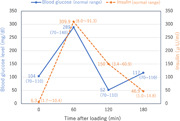

## BACKGROUND

Diabetes is one of the most common adverse effects of clozapine, a drug used to treat schizophrenia. If a patient with schizophrenia with normal glucose tolerance takes clozapine and subsequently develops diabetes, he/she will show increased insulin resistance and consequently hyperglycemia. If the patient develops hyperglycemia due to abnormal glucose tolerance, it is difficult for medical professionals to recognize that the patient is hyperglycemic unless he/she exhibits polyuria and thirst, therefore regular blood glucose testing is mandatory for patients receiving clozapine in Japan. In contrast, increased insulin resistance can lead to excessive insulin secretion and reactive hypoglycemia after meals. Thus, it is unknown whether hypoglycemia, rather than hyperglycemia, is a sign of impaired glucose tolerance. Clinical manifestations of hypoglycemia include autonomic symptoms such as cold sweats, limb tremors, numbness, nausea, vomiting, palpitations, tachycardia, and hunger; however, it is difficult to notice that these symptoms are caused by hypoglycemia unless the patients are using antidiabetic drugs such as hypoglycemic drugs or insulin. Although patients under treatment for diabetes are usually briefed about hypoglycemia symptoms, non‐diabetic patients have limited knowledge of hypoglycemia symptoms and it might be difficult for them to guess that their symptoms are caused by hypoglycemia. In addition, patients not using diabetes medications or insulin generally do not present with hypoglycemia, making it difficult for healthcare providers to suspect hypoglycemia. For example, if a patient complains of nausea, the healthcare provider first considers a digestive problem. If hypoglycemic symptoms appear as psychiatric symptoms such as anxiety, it is even more difficult for the healthcare provider to suspect hypoglycemia.

To the best of our knowledge, this is the first report on reactive hypoglycemia due to clozapine‐induced glucose intolerance in patients with schizophrenia.

## CASE PRESENTATION

The case was a 50‐year‐old man, born and raised as a second child to a sibling. He graduated from a local high school, with moderate academic performance. Subsequently, he entered a private university. When the patient was 22 years old, he developed hallucination and delusion. He visited Hospital A, where he was diagnosed with schizophrenia and was treated with antipsychotic medications. However, the psychiatric symptoms improved slightly, and he was admitted to Hospital A for medical care and protection. On admission, he was withdrawn from the university. He was treated an outpatient, but he relapsed several times and was hospitalized 10 times. The patient currently lives with his elder sister.

In February X − 1, the patient was admitted to Hospital A for medical care and protection because he was acting impulsively, verbally abusing his sister and threatening to kill her with a knife, in accordance with his hallucinations and delusions. Having met the diagnostic criteria for treatment‐resistant schizophrenia, clozapine treatment was initiated in May. In September, the dosage of clozapine was gradually increased to 500 mg; auditory hallucinations and delusions of persecution reduced, and verbal abuse and violent behavior disappeared.

In January of the following year (X), the patient's blood glucose level was measured because of increased complaints of postprandial fatigue. The postprandial blood glucose level was low. To address the hypoglycemia, 20 g of glucose was added to each meal; however, the postprandial blood glucose remained at approximately 40 mg/dl and did not improve. In February X, the patient was transferred to the psychiatric ward of our hospital for proper examination.

**Figure 1:**
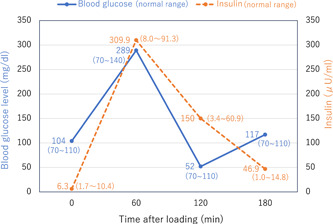
Result of oral glucose tolerance test (75 g OGTT) showing blood glucose and insulin levels. The normal range of each data is noted in brackets. The results show an abnormal increase in blood glucose and insulin followed by hypoglycemia.

On admission, the patient was mildly obese with a height of 170.0 cm, weight of 76.7 kg, and BMI of 26.5. There were no abnormalities in blood pressure, pulse rate, or on neurological examination. He was placed on 500 mg of clozapine, 30 mg of metoclopramide, and 10 mg of Lemborexant. The differential diagnoses of causes of hypoglycemia in the patient were insulinoma, insulin autoimmune syndrome, insulin antagonist hormone depletion, alcohol‐induced hypoglycemia, extra‐pancreatic tumor‐associated hypoglycemia, and reactive hypoglycemia. He consulted the Department of Diabetes Medicine of our hospital. He had a blood glucose level of 165 mg/dl, insulin 54.3 μU/ml (reference value: 0–18.7 μU/ml), and C‐peptide 7.9 ng/ml (reference value 0.8–2.5 ng/ml), which were high. There were no other significant abnormalities in blood counts or biochemical tests. Insulin antagonist hormones (growth hormone, somatomedin C, and cortisol) were within normal limits, and insulin antibodies were negative. Urine storage test showed that C‐peptide was within normal range. A glucose tolerance test (75 g OGTT) was performed (Figure 1). The results showed a blood glucose level of 104 mg/dl before glucose load, which increased to 289 mg/dl at 60 min after the glucose load, and then gradually decreased to 52 mg/dl at 120 min after the glucose load. At 60 min after glucose loading, insulin secretion exceeded 309 µU/ml.

In this case, blood tests showed hypoglycemia and excessive insulin secretion, leading to insulinoma and reactive hypoglycemia as differential diagnoses. Since hypoglycemia occurred only after meals and there were no abnormalities on abdominal imaging studies, the final diagnosis was reactive hypoglycemia underlying impaired glucose tolerance. We suggested that the hypoglycemia should be treated with diet and alpha‐glucosidase inhibitor (voglibose). He was transferred back to Hospital A on the 18th day after admission, where voglibose was initiated, the clozapine dose was adjusted, and hypoglycemia did not occur.

## DISCUSSION

In this case, according to an analysis of 9635 clozapine‐treated patients with the Clozaril Patient Monitoring Service in Japan,^1^ the three most frequent cause of clozapine discontinuation were neutropenia (5%), gastrointestinal tract (0.9%), and glucose intolerance (0.7%).

Type 2 diabetes, an adverse effect of clozapine, may result in abnormal glucose tolerance or reactive hypoglycemia as a prodromal symptom. Normally, an appropriate amount of insulin is secreted at the appropriate time after a meal to prevent an increase in postprandial blood glucose, but in reactive hypoglycemia, delayed insulin secretion after a meal causes hyperglycemia, resulting in excessive insulin secretion in response to high blood glucose, which in turn causes hypoglycemia.

Melkersson et al. suggested that the putative mechanism of clozapine‐induced reactive hypoglycemia is that clozapine induces insulin hypersecretion in vitro.^2^ Smith et al. reported that activation of the Akt/PKB signaling pathway in the brain of clozapine‐exposed rats was associated with hyperinsulinemia.^3^ To date, the rationale for individual and racial differences in clozapine‐induced reactive hypoglycemia is unclear.

To the best of our knowledge, there are no reports on clozapine‐induced hypoglycemia, but there are case reports of the development of hypoglycemia with other antipsychotics^4^, ^5^, ^6^, ^7^, ^8^, ^9^, ^10^, ^11^, ^12^, ^13^ (Table 1). Most patients develop hypoglycemia induced by second‐generation antipsychotics, with olanzapine and quetiapine being the most frequent. In addition, seven of the 11 cases were reported in Japanese patients. The considerations regarding the mechanisms of hypoglycemia in each case report can be summarized into five main categories: (i) inhibition of normal regulatory mechanisms of hypoglycemia by adrenergic alpha2‐receptor antagonism; (ii) atypical antipsychotics involve both the serotonergic and adrenergic pathways, causing hypoglycemia; (iii) atypical antipsychotics antagonize muscarinic receptors, resulting in continued basal insulin secretion even after glucose levels return to normal; (iv) atypical antipsychotics are antagonistic to muscarinic receptors, resulting in continued basal insulin secretion after glucose levels return to normal (this implies that antipsychotics inhibit ATP‐sensitive potassium channels in pancreatic beta cells, thereby suppressing glucose‐stimulated insulin secretion; (v) multiple receptor‐acting antipsychotics may interact with histamine H1, adrenergic alpha1, muscarinic M1, 5‐HT2A, 5‐HT7, and dopamine D2 and D3receptors, resulting in increased basal insulin secretion and hypoglycemia). These hypotheses may partially explain the pathophysiology of antipsychotic‐induced hypoglycemia, but further studies are required to fully elucidate this pathophysiology.

**Table 1:** Hypoglycemia induced by antipsychotics.

Study	Diagnosis	Age	Antipsychotic	Outcome of hypoglycemia
Ochi, 2020	Schizophrenia	60	RIS 6 mg	Recovered after discontinuation of RIS
			OLZ 10 mg	Recovered after discontinuation of OLZ
			HPD 4.5～18 mg	No hypoglycemia
Couto, 2019	Schizophrenia	67	HPD 15 mg ＋100 mg intramascular	Recovered after discontinuation of HPD
Cho, 2019	Dementia	74	QTP 300 mg	Recovered after discontinuation of QTP
Fujita, 2018	Bipolar disorder	62	QTP 25 mg	Recovered after discontinuation of QTP
Nagamine, 2016	Schizophrenia	77	RIS 2 mg	Recovered after changing to blonanserin
Omi, 2016	Schizophrenia	42	PAL 12 mg	Partially improved after discontinuation of PAL
Suzuki, 2012	Schizophrenia	50	QTP 400 mg	Recovered after changing to blonanserin 16 mg
Mondal, 2012	Parkinson's disease and psychosis	72	ARP 10 mg	Recovered after discontinuation of ARP
Suzuki, 2009	Schizophrenia	27	QTP 400～600 mg	Clinically recovered but asymptomatic hypoglycemia was still present after changing to perospirone 36 mg
	Schizophrenia	53	OLZ 20 mg	Recovered after RIS was reduced to 3 mg
	Schizophrenia	32	OLZ 20 mg	No information
Nagamine, 2006	Schizophrenia	47	OLZ 10 mg	Recovered after discontinuation of OLZ
Budman, 2001	Tourette's syndrome (seven cases)	20‐44	OLZ 11.7 mg (SD6.7)	No information

Abbreviations: ARP, aripiprazole; HPD, haloperidole; OLZ, olanzapine; PAL, paliperidone; QTP, quetiapine; RIS, risperidone.

The treatment of impaired glucose tolerance includes diet, exercise, and oral hypoglycemic medications. An alpha‐glycosidase inhibitor (voglibose), which slows the absorption of sugar in the intestinal tract, has been used in Japan since 2009 to prevent type 2 diabetes in patients with impaired glucose tolerance associated with hypertension, dyslipidemia, obesity, or a family history of diabetes in the second degree. In a randomized, double‐blind study^14^ of 1780 Japanese subjects with impaired glucose tolerance, voglibose reduced the incidence of type 2 diabetes by 40.5% compared to placebo, therefore voglibose may be effective in improving hypoglycemia. A low‐carbohydrate, high‐protein, and high‐lipid diet was recommended as dietary therapy in our patient. After using an optimal dose of voglibose, the hypoglycemia disappeared, although it occurred almost every day prior to hospitalization. The patient continued to take clozapine, and his psychiatric symptoms remained stable. The previous physician had added sugar to the diet for hypoglycemia, but, given the pathophysiology, it would be advisable to avoid this approach, as it causes a rapid rise in blood glucose levels and further aggravates hypoglycemia.

## CONCLUSION

We report a case of repeated reactive hypoglycemia during treatment with clozapine, which improved with the concomitant use of voglibose. Under conditions of impaired glucose tolerance, hypoglycemia may be caused by excessive postprandial insulin secretion. Hypoglycemia can present with physical symptoms such as fatigue and dizziness; however, if the patient is not receiving diabetes treatment, it is difficult to detect whether these symptoms are suggestive of hypoglycemia. Therefore, these symptoms may be evaluated as complaints or symptoms of psychiatric disorders. During clozapine administration to patients with schizophrenia, it is necessary to monitor physical and psychiatric symptoms due to reactive hypoglycemia and hyperglycemia. If abnormal glucose tolerance is a concern, it should be promptly detected using blood or oral glucose tolerance tests. Early intervention for impaired glucose tolerance may prevent clozapine discontinuation due to diabetes or hyperglycemia.

## AUTHOR CONTRIBUTIONS

Fumihiko Kawai, Satsuki Watanabe, Hirotaka Ebihara, Naoki Shimizu, Shiori Oshima, Hiroshi Okai, and Hisatoshi Arai treated the patient. Fumihiko Kawai and Satsuki Watanabe drafted the manuscript. Yoshiko Murata and Koji Matsuo critically reviewed the draft and revised it. All authors approved the final version of the manuscript.

## CONFLICT OF INTERESTS STATEMENT

Koji Matsuo has received honoraria from Kyowa Yakuhin Kogyo, Janssen Pharma, Viatris, Meiji Seika Pharma, Eisai, Sumitomo Pharma, Yoshitomiyakuhin, Otsuka Pharmaceutical, Takeda Pharmaceutical and Lundbeck Japan, and research donations from Shionogi, Eisai and Mochida Pharmaceutical. Satsuki Watanabe has received speaker's honoraria from Eisai, UCB Japan and Daiichi Sankyo.

## ETHICS APPROVAL STATEMENT

This study was approved by the Ethics Committee of Saitama Medical University Hospital.

## PATIENT CONSENT STATEMENT

We obtained written informed consent and signed release from the patient for the publication of this report.

## CLINICAL TRIAL REGISTRATION

N/A.

## Data Availability

N/A.
